# The first Paratropididae (Araneae, Mygalomorphae) from Colombia: new genus, species and records

**DOI:** 10.3897/zookeys.830.31433

**Published:** 2019-03-14

**Authors:** Carlos Perafán, William Galvis, Fernando Pérez-Miles

**Affiliations:** 1 Sección Entomología, Facultad de Ciencias, Universidad de La República, Iguá 4225, Montevideo, Uruguay Universidad de La República Montevideo Uruguay; 2 Laboratorio de Aracnología & Miriapodología (LAM-UN), Instituto de Ciencias Naturales, Universidad Nacional de Colombia, Bogotá, Colombia Universidad Nacional de Colombia Bogotá Colombia

**Keywords:** Andean Region, bald legged spiders, Ecuador, Neotropics, new genus, new species

## Abstract

The family of mygalomorph spiders Paratropididae Simon, 1889 is here reported for the first time for Colombia, where it is represented by three genera (*Anisaspis*, *Paratropis*, *Stormtropis***gen. n.**) and eight species. One genus, *Stormtropis*, and six species constitute new taxa that are here diagnosed, described and illustrated. The geographical distribution of *Paratropispapilligera* FO Pickard-Cambridge, 1896 and *Paratropiselicioi* Dupérré, 2015 are also redescribed and expanded on the basis of new material examined. The diagnosis of the subfamily Paratropidinae, *Paratropis* Simon, 1889 and *Anisaspis* Simon, 1892 are emended including the variations of the new species. Likewise, a geographic distribution map for the entire family and a taxonomic key for the males of Paratropidinae are included. Other biogeographic, morphological, and taxonomic aspects are discussed.

## Introduction

Paratropididae Simon, 1889, known as bald legged spiders, is one of the most enigmatic groups of Mygalomorphae due to its cryptic habits, singular biology, and controversial phylogenetic position. Paratropidids constitute a small family of spiders, currently comprising four genera and eleven species, distributed in Mexico, Central America, Lesser Antilles, and northern South America except Colombia (Raven 1985, WSC 2018). Their presence in Venezuela, Mexico, and Ecuador has been recently reported by Bertani (2013), Valdez-Mondragón et al. (2014) and Dupérré (2015), respectively. Paratropidids are cursorial, medium to small-sized mygalomorphs, which hide themselves in the surface layers of the soil (West in Raven 1999); found in leaf litter and rotten logs, under rocks, moss, and burrows in ravines (personal observations). The main paratropidid morphological characteristic is the presence of a scaly cuticle adapted to adhere to soil particles (Raven 1985, Jocqué and Dippenaar-Schoeman 2006).

Formerly, Paratropididae was considered the sister-group of Theraphosidae into Theraphosoidea, and this group was related with Barychelidae in Theraphosoidina (Raven 1985, Goloboff 1993). However, results of some molecular and total evidence studies suggest a distant phylogenetic relationship of Paratropididae with the Theraphosoidina clade, not closely related with Theraphosidae or Barychelidae (Bond and Hedin 2006, Bond et al. 2012). Conversely, in recent studies, Paratropididae was recovered as sister group of some ctenizids (Wheeler et al. 2017), or some nemesiids (Garrison et al. 2016); although, both studies pointing out this position as questionable. Likewise, in Fernandez et al. (2018) Paratropididae was resolved as a sister group of *Macrothele* (Macrothelidae), but this study focused in Araneomorphae, and mygalomorph sampling was very limited. Hamilton et al. (2016) recovered Paratropididae as the sister group to all other Aviculariodea taxa, with the exception of *Bymainiella* sp. (Hexathelidae); this grouping was followed by Hedin et al. (2018).

Paratropidids are characterized by the soil encrusted or scaly cuticle, weakly or ascopulate tarsi I and II and absence of scopulae elsewhere, maxillary lobes elongated, and the presence of labial cuspules usually arranged in an anterior rectangular group (Raven 1985). Pérez-Miles et al. (2017) found that the putative scopula of paratropidids was in fact a pseudoscopula, constituted of chemosensory not-adhesive setae. The family comprises two subfamilies, Paratropidinae Simon, 1889 and Glabropelmatinae Raven, 1985. Paratropidinae is characterized by a long single tooth on the superior tarsal claws, the steeply elevated eye tubercle, very long anterior maxillary lobes, and absence of a tibial apophysis and claw tufts, unlike Glabropelmatinae that has thin claw tufts (without scopula), a bifurcated tibial apophysis, a typical elevated eye tubercle, and shorter anterior maxillary lobes (Raven 1985).

Paratropidinae includes three genera, *Paratropis* Simon, 1889 with six species, distributed in Mexico, Venezuela, Brazil and Peru; *Anisaspis* Simon, 1892 with one species from Saint Vincent island; and *Anisaspoides* FO Pickard-Cambridge, 1896 with one species from Brazil. *Anisaspis* and *Anisaspoides* are only known by females. Glabropelmatinae includes only one genus *Melloina* Brignoli, 1985, with three species, distributed in Venezuela and Panama (WSC 2018).

Two characteristics have been important in Paratropidinae taxonomy, the number of spinnerets, four (*Paratropis*) or two (*Anisaspis*, *Anisaspoides*), and the presence of a third tarsal claw, only on leg I (most *Paratropis*), only on legs I and II (*Anisaspoides*), or absent (*Anisaspis*) (Raven 1985, Pickard-Cambridge 1896, Valdez-Mondragón et al. 2014).

The presence of Paratropididae in Colombia was informally indicated by  de Mello-Leitão (1941) and in unpublished studies (Flórez-D. and Sánchez-C. 1995, Perafán et al. 2013); however, this is the first formal report for the country. A review of mygalomorph spiders from Colombia including field and collection data produced some important results for Paratropididae, which we present here: a new genus, *Stormtropis* gen. n., with four new species; a new species of *Anisaspis*, which represent the first male description for the genus and the first record for South America; one new species of *Paratropis*; and a new record of *P.papilligera* FO Pickard-Cambridge, 1896 and *P.elicioi* Duperré, 2015. We discuss the geographic distribution and diversity of paratropidids as well as some biological and morphological characteristics.

## Materials and methods

The general description format follows Raven (1999) and Bertani (2013), with modifications. Specimens were examined using an Advanced Optical stereomicroscope. Photographs were taken with an Olympus LC30 camera adapted to a stereomicroscope. All measurements are in millimeters and were taken with an ocular micrometer. Legs and palp measurements were taken along a dorsal longitudinal line of the left side. Total body length excludes chelicerae and spinnerets. Spination: we consider as spines the thick, sclerotized setae, with acute and non-translucent apex; similar setae but with translucent apex were not counted. Male palpal bulbs (usually left) were removed from cymbium for study and illustrations. Records without geographic coordinates were determined using GoogleMaps and the gazetteers GeoLocator and GeoNames. The distribution map was produced using the Geographic Information System QGIS ‘Girona’ (version 3.0, QGIS team, www.qgis.org), with raster files from NaturalEarth and DivaGis. WSC means World Spider Catalog.

Abbreviations in figures or text are as follows:

**ALE** anterior lateral eyes;

**AME** anterior median eyes;

**fe** femur;

**ITC** inferior tarsal claw;

**me** metatarsus;

**p** prolateral;

**pa** patella;

**pd** prolatero-dorsal;

**PME** posterior median eyes;

**PMS** posterior median spinnerets;

**PLE** posterior lateral eyes;

**PLS** posterior lateral spinnerets;

**pv** prolatero-ventral;

**r** retrolateral;

**rv** retrolatero-ventral;

**STC** superior tarsal claw;

**ta** tarsus;

**ti** tibia.

Examined materials are deposited in the following institutions:

**BMNH (NHM)**Natural History Museum, London;

**ICN**Instituto de Ciencias Naturales, Universidad Nacional de Colombia, Bogotá;

**FC-My**Facultad de Ciencias, Universidad de la República, Montevideo, Uruguay;

**QCAZ**Quito-Católica-Zoología, Museo de Zoología, Pontificia Universidad Católica del Ecuador.

## Taxonomy

### Family Paratropididae Simon, 1889

#### Subfamily Paratropidinae Simon, 1889

**Emended diagnosis.**Paratropidinae spiders differ from those of Glabropelmatinae (that includes only the genus *Melloina* Brignoli, 1985) by the absence of claw tufts, presence of a single long tooth on the superior tarsal claws (STC), the steeply elevated eye tubercle, and the book lung apertures projected, oval and sclerotized. Males without tibial apophysis (*Anisaspis*, *Paratropis* and *S.muisca* sp. n.) or composed by a single prolateral branch (*Stormtropis* gen. n., except *S.muisca* sp. n.). *Anisaspoides* males are still unknown.

**Included genera.***Anisaspis* Simon, 1892, *Anisaspoides* FO Pickard-Cambridge, 1896, *Paratropis* Simon, 1889 and *Stormtropis* Perafán, Galvis and Pérez-Miles gen. n.

#### Identification key to males of Paratropidinae species

**Table:** 

1	One pair of spinnerets (PLS) (Figure 1B) and all legs with only two tarsal claws (only STC)	***Anisaspiscamarita* sp. n.**
–	Two pairs of spinnerets (PMS and PLS), legs with or without inferior tarsal claw (ITC)	**2**
2	Palpal bulb with embolus relatively straight, thin and elongated. Leg I without tibial apophysis	**3 *Paratropis***
–	Palpal bulb pyriform elongated, embolus slightly curved tapering to the apex and with a subapical triangular tooth. Leg I with or without tibial apophysis	**5 *Stormtropis* gen. n.**
3	Palpal bulb with short embolus and slightly sigmoid (Valdez-Mondragón et al. 2014, figs 10–15)	*** Paratropis tuxtlensis ***
–	Palpal bulb with large embolus	**4**
4	Palpal bulb with very long and straight embolus (Figure 2E, F), tibia I without basal retrolateral conic process	*** Paratropis elicioi ***
–	Palpal bulb with very thin and long embolus, distally curved (Figure 4F, G), and tibia I with a basal retrolateral conic process with spiniform setae (Figure 4D, E)	*** Paratropis papilligera ***
5	Leg I without tibial apophysis. Palpal bulb as Figure 6D, E	***Stormtropismuisca* sp. n.**
–	Leg I with tibial apophysis	**6**
6	Discontinuous row and few cheliceral teeth on promargin (2-2-3) (Figure 9B), tibial apophysis with a long base and separated from the tibia and with few spines (Figure 9D, E)	***Stormtropisparvum* sp. n.**
–	Continuous row and numerous cheliceral teeth on promargin, tibial apophysis with a short base and with many spines	**7**
7	Tibial apophysis with numerous spines on the proximal row (12) (Figure 7D, E); presence of a sclerotized dark mark on proximal dorsal tibia, with a slight excavation (Figure 7A)	***Stormtropispaisa* sp. n.**
–	Tibial apophysis with less spines on the proximal row (6) (Figure 5D, E); without sclerotized dark mark on tibia (Figure 5A)	***Stormtropiscolima* sp. n.**

##### Anisaspis

Taxon classificationAnimaliaAraneaeParatropididae

Simon, 1891

Figure 1

###### Type species.

*Anisaspistuberculata* Simon, 1892, deposited in NHM, examined.

###### Diagnosis.

**Figure 1.**
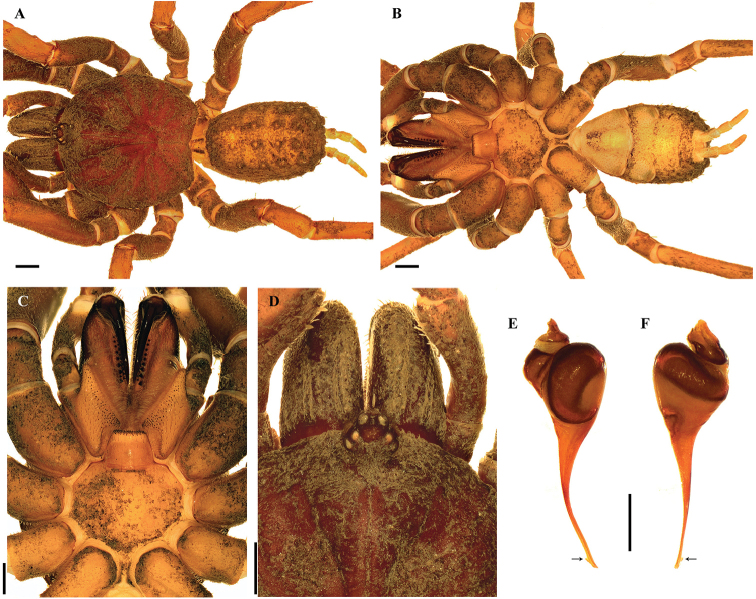
*Anisaspiscamarita* sp. n., male. **A, B***habitus***A** dorsal view **B** ventral view **C** sternum, labium and maxillae **D** caput and ocular tubercle **E, F** palpal bulb **E** prolateral view **F** retrolateral view. Arrow points to the triangular tooth on the subapical region of the embolus. Scale bars: 1.0 mm (**A–D**); 0.5 mm (**E, F**).

*Anisaspis* differs from other paratropidid genera by the presence of only two spinnerets (PLS) (Figure 1D) and all legs with only two tarsal claws (STC), males without tibial apophysis and palpal bulb with sinuous embolus (Figure 1E, F). PLS relatively long (Figure 1A, B). Females remain unknown.

###### Remarks.

The specimen described as holotype female of *A.tuberculata* is actually a juvenile specimen. Therefore, females of this genus remain unknown.

###### Included species.

*Anisaspistuberculata* Simon, 1892 and *Anisaspiscamarita* Perafán, Galvis & Pérez-Miles, sp. n.

###### Distribution.

Lesser Antilles, Saint Vincent island and Colombia, on the eastern flank of the Eastern Cordillera, Meta Department, Llanos foothills (Figure 10); from sea level to 570 m altitude.

##### Anisaspis camarita sp. n.

Taxon classificationAnimaliaAraneaeParatropididae

http://zoobank.org/E0378E15-1F64-4F82-BE43-3305D962984C

Figure 1

###### Type material.

***Holotype*** male from Colombia, Meta, Villavicencio, Bosque de Bavaria, 4.18089N, 73.64800W, 570 m, 7-X-2005, col. HJ Salazar (ICN-Ar 1404).

###### Diagnosis.

*Anisaspiscamarita* sp. n. differs from *A.tuberculata* by the absence of a spine on dorsal tarsi distally, longer spinnerets (PLS) with apical segment digitiform (domed in *A.tuberculata*) (Figure 1A, B) and AME on a super-tubercle (another higher tubercle on the ocular tubercle, Figure 1D).

###### Description.

***Holotype male*** (ICN-Ar 1404) (Figure 1): total length 12.6; carapace length 6.0, width 6.5; abdomen length 5.8, width 3.4; chelicerae length 2.8. Color (in alcohol): body with soil particles encrusted; carapace, chelicerae, coxa, trochanter and femur dark reddish brown; abdomen dorsally and patella-tarsus brown. Carapace: glabrous, striae conspicuous, lateral margins with single line of curved setae mixed with disperse clubbed setae; caput strongly arched, separated from thoracic region by transverse shallow fovea, straight, width 1.1 (Figure 1A). Eyes and ocular tubercle: tubercle length 1.0, width 1.0, very elevated (height 0.8) and forwardly directed, with few stout setae. AME on a supertubercle (another higher tubercle on the ocular tubercle, Figure 1D). Clypeus absent. Anterior eye row slightly procurved, posterior recurved. Ocular sizes and interdistances: AME 0.25, ALE 0.30, PME 0.23, PLE 0.28, AME-AME 0.15, AME-ALE 0.05, PME-PME 0.48, PME-PLE 0.05, ALE-PLE 0.08, AME-PME 0.10, ALE-ALE 0.65, PLE-PLE 0.70. Chelicerae: short sparse bristles on dorsal and lateral areas, long fine bristles on ventral and anterior area. Rastellun absent. Cheliceral furrow with two rows of teeth well-developed, 12 and 9 teeth on promargin and retromargin, respectively. Fang long. Labium: length 0.7, width 0.9, with 77 cuspules on anterior edge (Figure 1C). Labio-sternal groove narrower in the middle than laterally. Maxillae longer than wide, with the anterior prolateral lobe very elongated, conical (Figure 1C); with 75/78 rounded cuspules spaced, largely spread over prolatero-ventral border from the inner edge to anterior lobe. Lyra absent. Sternum: length 2.5, width 3.4; three pairs of sigillae, anterior and median subcircular, posterior sigillae oval, all submarginal. Anterior half of sternum elevated (Figure 1C).

Legs: cuticle with soil particles encrusted. Leg and palpal segments measurements provided in Table 1. Leg I clearly thicker than the others. Bristles, plumose and thorn-like setae and spines evident. Trichobothria: filiform, on central 2/3 of tarsi, palp 5, leg I 8, II 7, III 6, IV 8; on distal 1/4 of metatarsi, legs I-III 4, IV 5; and on proximal 1/3 of tibiae, palp two rows of 3 each, legs I-III two rows of 4 each, IV two rows of 4 and 1 respectively. Scopula absent. Pseudoscopula weak and divided by conical longer setae, only present on distal tarsi I and II; tarsi III and IV with few sparse pseudoscopula setae. Claw tufts absent. Tarsal claws: ITC absent on all legs but a very small tooth present on right leg I in the same position of ITC; STC with one medial tooth on all legs. Tibial apophysis absent. Spination: principally thorn-like setae on all segments. Spines: palp and legs I-II 0; leg III, fe 0, pa 0, ti 0, me 1pd, 3v, ta 1p; leg IV, fe 0, pa 0, ti 0, me 2v, ta 1p.

**Table 1.** Lengths (in mm) of legs and palpal segments of the holotype male *Anisaspiscamarita* sp. n.

	I	II	III	IV	Palp
Femur	6.4	5.3	4.6	6.1	2.6
Patella	3.3	2.5	2.2	2.4	1.5
Tibia	6.0	4.2	3.4	5.1	1.9
Metatarsus	5.0	4.5	4.0	5.9	–
Tarsus	2.0	2.0	2.0	2.4	1.0
Total	22.7	18.5	16.2	21.9	7.0

Palp: cymbium with two unequal lobes separated by a sclerotized groove; tibia with shallow distoventral groove. Palpal bulb pyriform elongated; embolus curved, long, tapering to the apex, apex wide; a triangular translucent tooth on the subapical region, close to the apex (Figure 1E, F).

Abdomen: with four longitudinal dorsal rows of seven small tubercles, each emitting from its summit a plumose, bacilliform seta; lateral area finely tuberculate, with smaller plumose setae (Figure 1A). Book lung apertures projected, oval, sclerotized (Figure 1B). Spinnerets: PMS absent; PLS length 2.8, apical segment digitiform. Basal segment of PLS divided in two unequal cuticle plates (Figure 1B).

***Female.*** Unknown.

###### Distribution.

Only known from its type locality, in the foothills of the Eastern Cordillera of Colombian Andes (at 570 m altitude), Meta Department, Bavaria forest (Figure 10).

###### Etymology.

The specific epithet *camarita* is a noun in apposition which means friend, in the colloquial way of the Llanos Region of Colombia, where this species is distributed.

##### Paratropis

Taxon classificationAnimaliaAraneaeParatropididae

Simon, 1889

Figs 2 , 3 , 4

###### Type species.

*Paratropisscruposa* Simon, 1889.

###### Diagnosis.

**Figure 2.**
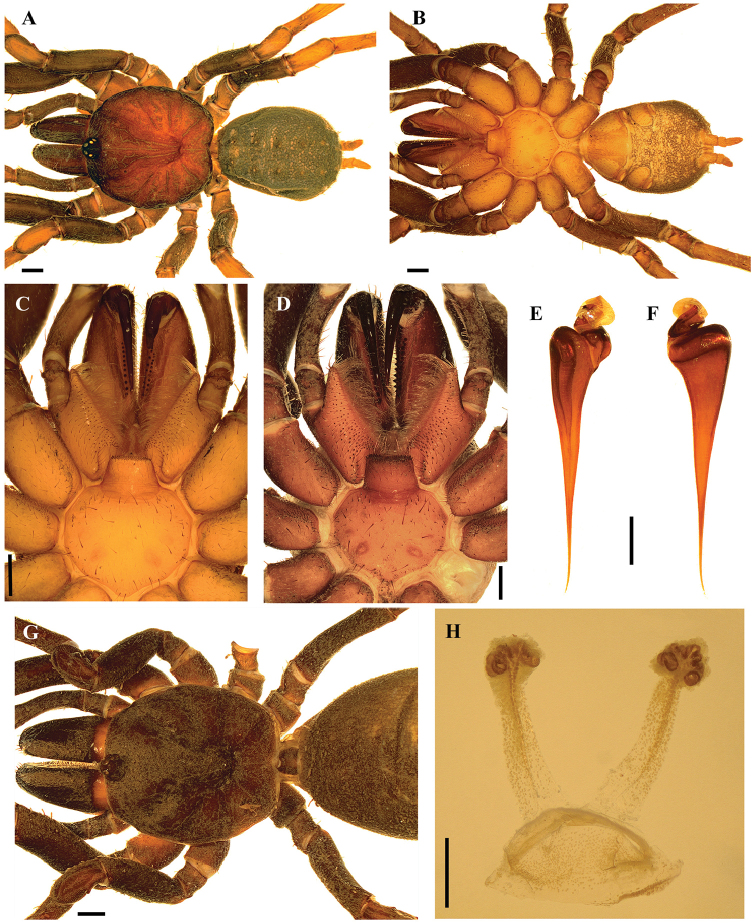
*Paratropiselicioi* Dupérré, 2015. **A–C** male **A, B***habitus***A** dorsal view **B** ventral view **C** sternum, labium and maxillae **D** sternum, labium and maxillae female **E, F** palpal bulb **E** prolateral view **F** retrolateral view **G***habitus* female, dorsal view **H** spermathecae. Scale bars: 1.0 mm(**A–D, G**); 0.5 mm (**E–H**).

**Figure 3.**
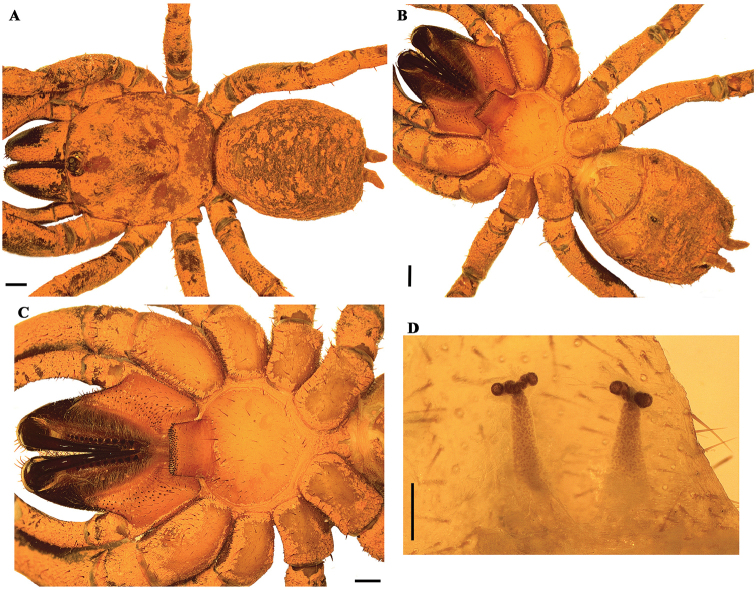
*Paratropisflorezi* sp. n., female. **A, B***habitus***A** dorsal view **B** ventral view **C** sternum, labium and maxillae **D** spermathecae. Scale bars: 1.0 mm (**A–C**); 0.5 mm (**D**).

**Figure 4.**
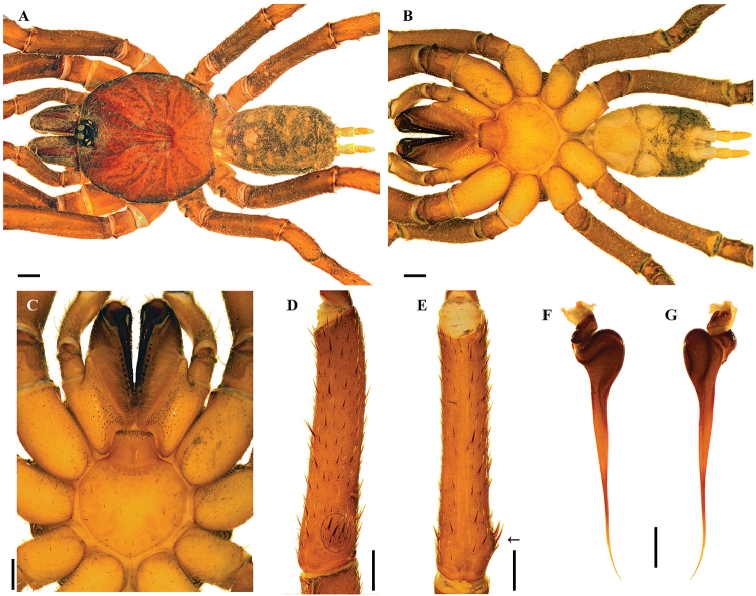
*Paratropispapilligera* FO Pickard-Cambridge, 1896, male. **A, B***habitus***A** dorsal view **B** ventral view **C** sternum, labium and maxillae **D, E** tibia I **D** retrolateral view **E** ventral view **F, G** palpal bulb **F** prolateral view **G** retrolateral view. Arrow and circle indicate the basal retrolateral conic process on tibia I. Scale bars: 1.0 mm (**A–C**); 0.5 mm (**F, G**).

*Paratropis* differs from other paratropidids by the combination of the following characters: presence of ITC on legs I, two pairs of spinnerets (PMS and PLS) (Figs 2B, 3B, 4B), males without tibial apophysis, and palpal bulb with embolus relatively straight, thin and very elongated (Figs 2E, F, 4F, G); females with spermathecal receptacles with multilobed fundus (Figs 2H, 3D).

###### Included species.

*Paratropiselicioi* Dupérré, 2015, *Paratropisflorezi* Perafán, Galvis & Pérez-Miles, sp. n., *Paratropispapilligera* FO Pickard-Cambridge, 1896, *Paratropissanguinea* Mello-Leitão, 1923, *Paratropisscruposa* Simon, 1889, *Paratropisseminermis* Caporiacco, 1955, *Paratropistuxtlensis* Valdez-Mondragón, Mendoza & Francke, 2014.

###### Distribution.

Mexico, Lesser Antilles, and northern South America (Brazil, Colombia, Ecuador, Peru and Venezuela). In Colombia it is widely distributed in the three mountain ranges that make up the Andes, the inter-Andean valleys and lowlands of the Amazon, Llanos, and Caribbean regions (Perafán 2017) (Figure 10).

##### Paratropis elicioi

Taxon classificationAnimaliaAraneaeParatropididae

Dupérré, 2015

Figure 2

###### Type material.

***Holotype*** male from Ecuador, Cotopaxi Province, Otonga Biological Reserve, near Rio Esmeraldas, 0.41941S, 78.99607W, 1717 m, 25.xi–08.xii.2014, pitfall, col. N Dupérré & E Tapia (QCAZ), not examined.

###### Additional material Examined.

Colombia, Nariño, Barbacoas, Altaquer, Reserva Natural Río Ñambí, 1.3N, 78.08333W, 1400 m, 17-27-vii-2012, col. M Medrano, A García, Y Cifuentes, D Martínez, 1 male (ICN-Ar 11435); one female with the same data (ICN-Ar 11436); one female from the same locality, 1440 m, 17-30-vi-2011, col. A García (ICN-Ar 6974). Ecuador: Pichincha: Santo Domingo, 466 m, 1-xi-1999, col. M Rivadeneira, 1 female (QCAZ, MV-PAR-018); Nanegalito, 1500 m, 27-xii-1996, col. M Davalos, 1 female (QCAZ, MV-PAR-015); Nanegalito, 1400 m, 23-i-1993, col. C Segovia, 1 female (QCAZ, MV-PAR-07); Las Tolas, 20-iii-1989, col. V Utreras, 1 female (QCAZ, MV-PAR-031).

###### Emended diagnosis.

Males of *P.elicioi* differ from those of other *Paratropis* species by the morphology of the palpal bulb with very long and straight embolus (Figure 2E, F). Females can be distinguished by the morphology of spermathecal receptacles with long neck, longitudinal dorsal fold and numerous apical lobes (Figure 2H).

###### Remarks.

The examination of new material from Colombia and Ecuador allowed us to infer that both the diagnosis and the descriptions of the two sexes of *P.elicioi* were inaccurate (Dupérré 2015). Below we present an emended description.

###### Description.

***Male*** (ICN-Ar 11435) (Figure 2A–C, E, F): total length 13.0; carapace length 6.1, width 6.1; abdomen length 6.0, width 3.8; chelicerae length 1.9. Color (in alcohol): body with few soil particles encrusted; carapace, chelicerae and legs reddish brown; abdomen grayish brown (Figure 2A). Carapace: slightly setose, with a single line of curved setae mixed with disperse clubbed setae, striae conspicuous; caput arched, separated from thoracic region by transverse shallow fovea, straight, width 0.9 (Figure 2A). Eyes and ocular tubercle: tubercle length 0.9, width 1.1, very elevated (height 0.8) and forwardly directed, with few setae. Clypeus absent. Anterior eye row slightly procurved, posterior recurved. Ocular sizes and interdistances: AME 0.35, ALE 0.35, PME 0.20, PLE 0.25, AME-AME 0.10, AME-ALE 0.03, PME-PME 0.50, PME-PLE 0.03, ALE-PLE 0.08, AME-PME 0.03, ALE-ALE 0.65, PLE-PLE 0.70. Chelicerae: short sparse bristles on dorsal and lateral areas, long fine bristles on ventral and anterior area; basal segment with clubbed-plumose setae. Rastellum absent. Cheliceral furrow with two rows of teeth well developed, 16/14 and 11/13 teeth on promargin and retromargin, respectively. Labium: length 0.7, width 1.4, with 75 cuspules on anterior edge (Figure 2C). Labio-sternal groove with two lateral mounds. Maxillae longer than wide, with the anterior prolateral lobe very elongated, conical (Figure 2C); with 73/76 cuspules spaced, largely spread over prolatero-ventral border from the inner edge to anterior lobe. Lyra absent. Sternum: length 2.5, width 3.2; three pairs of sigillae, anterior subcircular, median and posterior sigillae oval; anterior and median sigillae marginal, posterior submarginal (Figure 2C).

Legs: cuticle normal. Leg and palpal segments measurements provided in Table 2. Leg I clearly thicker than the others. Bristles, clubbed and thorn-like setae present. Trichobothria: filiform, on central 2/3 of tarsi, palp 4, leg I 9, II 9, III 8, IV 9; on distal 1/4 of metatarsi, leg I 5, II 5, III 4, IV 4; on proximal 1/3 of tibiae, palp with two rows of 3 and 4 respectively, legs I–III two groups of 4 each, IV 2r-5p-1d (proximal/distal). Scopula absent. Pseudoscopula weak and divided by conical longer setae, only present on distal tarsi I, II and III. Claw tufts absent. Tarsi distally incrassate on anterior legs. Tarsal claws: ITC present on leg I; STC with one tooth on all legs. Spination: principally thorn-like setae on all segments. Spines: palp 0; leg I 0; leg II 0; leg III fe 0, pa 0, ti 0, me 1pv, ta 1pv; leg IV fe 0, pa 0, ti 0, me 1pv, ta 2pv.

**Table 2.** Lengths (in mm) of legs and palpal segments of male (ICN-Ar 11435) / female (ICN-Ar 11436) *Paratropiselicioi*.

	I	II	III	IV	Palp
Femur	6.0/6.0	4.9/4.3	4.3/4.0	5.8/5.7	2.9/3.3
Patella	3.0/3.2	2.3/2.5	2.1/2.5	2.3/2.8	1.7/1.8
Tibia	5.2/5.0	3.8/3.5	2.9/3.0	5.1/5.1	2.0/2.2
Metatarsus	4.9/4.0	4.3/3.3	3.9/3.6	5.7/5.4	–
Tarsus	2.4/1.7	2.2/1.7	2.0/1.8	2.1/2.5	0.9/2.5
Total	21.5/19.9	17.5/15.3	15.2/14.9	21.0/21.5	7.5/9.8

Palp: cymbium with two unequal lobes separated by a sclerotized groove; tibia with distoventral groove. Palpal bulb pyriform very elongated; embolus as long as tibia and half patella, straight then tapering to the apex, apex stout but flattened (Figure 2E, F).

Abdomen: with four longitudinal dorsal rows of seven small tubercles, each emitting from its summit a plumose, bacilliform seta. Book lung apertures projected, oval, sclerotized (Figure 2B). Spinnerets: PMS 0.50; PLS length 1.9, apical segment digitiform. Basal segment of PLS divided in two unequal cuticle plates (Figure 2B).

***Female*** (ICN-Ar 6974) (Figure 2D, G, H): total length 18.5, carapace length 7.2, width 7.1; abdomen length 10.0, width 7.0; chelicerae length 4.0. Color (in alcohol): body with soil particles encrusted; carapace, and legs reddish dark brown, chelicerae dark brown, abdomen grayish brown (Figure 2G). Carapace: slightly setose, lateral margins with single line of spiniform setae with a single line of curved setae mixed with disperse clubbed setae; striae conspicuous; caput arched, separated from thoracic region by transverse fovea, straight, width 1.4 (Figure 2G). Eyes and ocular tubercle: tubercle length 1.2, width 1.3, very elevated (height 0.9) and forwardly directed, with few setae. Clypeus absent. Anterior eye row slightly procurved, posterior recurved. Ocular sizes and interdistances: AME 0.35, ALE 0.38, PME 0.28, PLE 0.33, AME-AME 0.18, AME-ALE 0.05, PME-PME 0.65, PME-PLE 0.03, ALE-PLE 0.05, AME-PME 0.05, ALE-ALE 0.73, PLE-PLE 0.85. Chelicerae: short sparse bristles on dorsal and lateral areas, long fine bristles on ventral and anterior area; basal segment with clubbed plumose setae. Rastellum absent. Cheliceral furrow with two rows of teeth well-developed, 13/14 and 13/12 teeth on promargin and retromargin, respectively. Labium: length 1.1, width 1.8, with 97 cuspules on anterior edge (Figure 2D). Labio-sternal groove with two lateral mounds. Maxillae longer than wide, with the anterior prolateral lobe very elongated, conical (Figure 2D); with 91/97 cuspules spaced, largely spread over prolatero-ventral border from the inner edge to anterior lobe. Lyra absent. Sternum: length 2.8, width 3.9; three pairs of sigillae, anterior subcircular, median and posterior sigillae oval; anterior and median sigillae marginal, posterior submarginal. Anterior edge of sternum with a semicircular area slightly elevated (joined to labio-sternal groove) (Figure 2D).

Legs: cuticle normal. Leg and palpal segments measurements provided in Table 2. Leg I clearly thicker than the others. Bristles, thorn-like setae and spines present. Trichobothria: filiform, on central 2/3 of tarsi, palp 9, leg I 11, II 10, III 9, IV 8; on distal 1/4 of metatarsi, leg I 5, II 4, III 4, IV 5; on proximal 1/3 of tibiae, palp 7 in two groups of 4 and 3 respectively, legs I-III two groups of 4 each, IV 2r-5p-1d (proximal/distal). Scopula or pseudoscopula absent. Claw tufts absent. Tarsal claws: ITC present on leg I; STC with one tooth on all legs. Spination: principally thorn-like setae on all segments. Spines: palp fe 0, pa 0, ti 0, ta 1pv, 2rv; leg I fe 0, pa 0, ti 0, me 5pv, 10rv, ta 8pv, 7rv; leg II fe 0, pa 0, ti 0, me 1pd, 2pv, 1r, 3rv, ta 1rv; leg III fe 0, pa 0, ti 0, me 2p, 3pv, 1r, 2rv, ta 1pv, 1rv; leg IV fe 0, pa 0, ti 0, me 3pv, 1rv, ta 2pv.

Abdomen: with four longitudinal dorsal rows of seven small tubercles, each emitting from its apex a plumose, bacilliform seta. Book lung apertures projected, oval, sclerotized. Two spermathecal receptacles with a long neck, with a longitudinal dorsal fold, ended in a multilobed fundus (Figure 2H). Spinnerets: PMS length 0.60; PLS length 3.0, apical segment digitiform. Basal segment of PLS divided in two unequal cuticle plates.

###### Distribution.

South of Colombia and north of Ecuador, on the Western Andean montane forest, between 500–1700 m altitudes. In Colombia it’s distributed on Nariño Department (Barbacoas, Reserva Natural Río Ñambí) and Ecuador distributed on Cotopaxi Province (Otonga Biological Reserve) and Pichincha Province (Santo Domingo; Nanegalito; Las Tolas) (Figure 10).

##### Paratropis florezi sp. n.

Taxon classificationAnimaliaAraneaeParatropididae

http://zoobank.org/260E4931-0972-4FB1-8BEE-68CC7A4D401B

Figure 3

###### Type material.

***Holotype*** female from Colombia, Valle del Cauca, km 16 road Cali-Buenaventura, 3.52519N, 76.61992W, 1800 m, 14-xii-2014, col. C Perafán (ICN-Ar 11437). ***Paratypes***: three females with the same data (ICN-Ar 11438).

###### Diagnosis.

Females of *Paratropisflorezi* sp. n. differ from those of all other species of *Paratropis* by having the abdominal tubercles not prominent (Figure 3A) and by the spermathecal receptacles with a long neck, with the base widened and apex narrow, ended in a fundus with several projected lobes (Figure 3D).

###### Description.

***Female*** (ICN-Ar 11437) (Figure 3): total length 15.2, carapace length 7.0, width 6.7; abdomen length 7.2, width 5.6; chelicerae length 3.4. Color (in alcohol): body with soil particles encrusted; carapace and legs reddish dark brown, chelicerae dark brown, abdomen grayish brown (Figure 3A). Carapace: scarcely setose, lateral margins with single line of spiniform setae; striae conspicuous; caput arched, separated from thoracic region by transverse fovea, slightly procurved, width 1.3 (Figure 3A). Eyes and ocular tubercle: tubercle length 0.8, width 0.7, very elevated (height 0.8) and slightly forwardly directed, with few setae. Clypeus absent. Anterior eye row slightly procurved, posterior recurved. Ocular sizes and interdistances: AME 0.38, ALE 0.35, PME 0.18, PLE 0.33, AME-AME 0.10, AME-ALE 0.08, PME-PME 0.55, PME-PLE 0.05, ALE-PLE 0.10, AME-PME 0.05, ALE-ALE 0.80, PLE-PLE 0.83. Chelicerae: short sparse bristles on dorsal and lateral areas, long fine bristles on ventral and anterior area. Rastellum absent. Cheliceral furrow with two rows of teeth well-developed, 11/13 and 9/12 teeth on promargin and retromargin, respectively. Labium: sub-rectangular, length 1.0, width 1.8, with 120 cuspules on anterior edge (Figure 3C). Labio-sternal groove with two lateral mounds. Maxillae longer than wide, with the anterior prolateral lobe very elongated, conical (Figure 3C); with 80/71 cuspules spaced, largely spread over prolateral-ventral border from the inner edge to anterior lobe. Sternum: heart shaped length 2.9, width 3.8; three pairs of sigillae, anterior subcircular, median and posterior sigillae oval; anterior and median sigillae marginal, posterior submarginal. Anterior edge of sternum with a semicircular area slightly elevated (joined to labio-sternal groove) (Figure 3C).

Legs: cuticle with soil particles encrusted. Leg and palpal segments measurements provided in Table 3. Palp, tarsi swollen. Leg I, clearly thicker than the others. Bristles, thorn-like setae, and spines present. Trichobothria: filiform, on central 2/3 of tarsi, palp 9, leg I 12, II 11, III 9, IV 13; on distal 1/4 of metatarsi, leg I 5, II 4, III 5, IV5; on proximal 1/3 of tibiae, palp and leg I two rows of 4 each, II two groups of 4 and 5 respectively, III two groups of 4 each, IV 2r-5p-1d (proximal/distal). Scopula or pseudoscopula absent. Claw tufts absent. Tarsal claws: ITC present on leg I; STC with one curved tooth on all legs. Spination: principally thorn-like setae on all segments. Spines: palp fe 0, pa 0, ti 0, ta 1pv, 4rv; leg I fe 0, pa 0, ti 0, me 10v, 5pv, 7rv, ta 10pv, 10rv; leg II fe 0, pa 0, ti 0, me 4v, 1pd, 1pv, 1rv, ta 3rv; leg III fe 0, pa 0, ti 0, me 3v, 2pd, 2pv, 1rv, ta 1pv, 1rv; leg IV fe 0, pa 0, ti 0, me 3v, 2pv, ta 5pv.

**Table 3.** Lengths (in mm) of legs and palpal segments of the holotype female *Paratropisflorezi* sp. n.

	I	II	III	IV	Palp
Femur	5.9	4.6	3.0	5.6	3.1
Patella	3.2	2.6	2.3	2.7	1.9
Tibia	4.5	3.0	2.4	4.6	1.9
Metatarsus	3.8	3.4	3.3	5.1	–
Tarsus	2.0	1.9	2.0	2.6	2.6
Total	19.4	15.5	13.0	20.6	9.5

Abdomen: with four longitudinal dorsal rows of seven small tubercles, each emitting from them a plumose, bacilliform seta. Book lung apertures projected, oval, sclerotized (Figure 3B). Two spermathecal receptacles with a long neck, with the base widened and apex narrow, ended in a fundus with several projected lobes (Figure 3D). Spinnerets: PMS length 0.6; PLS length 2.7, apical segment digitiform. Basal segment of PLS divided in two unequal cuticle plates (Figure 3B).

***Male.*** Unknown.

###### Distribution.

Only known from its type locality, in the western Cordillera of Colombian Andes, Valle del Cauca Department, km 16 road Cali-Buenaventura, at 1800 m altitude (Figure 10).

###### Natural history.

The females of *Paratropisflorezi* sp. n. live in shallow burrows that they dig in the substrate of the ravines of the road.

###### Etymology.

The species epithet is a noun in genitive, in honor of Dr Eduardo Flórez Daza (ICN), in recognition of his friendship, teachings, and vast contributions to Colombian arachnology.

##### Paratropis papilligera

Taxon classificationAnimaliaAraneaeParatropididae

FO Pickard-Cambridge, 1896

Figure 4

###### Type material.

***Holotype*** male and ***paratype*** female from Santarem, Pará, Brazil, deposited in NHM, only male examined.

###### Additional material examined.

Colombia, Amazonas, Leticia, km 11 road to Tarapacá, 100 m, 25-iv-2002, it was collected manually in the day on leaf litter, col. G Amat and Estudiantes Introducción Sistemática Animal - Universidad Nacional de Colombia, 1 male (ICN-Ar 2315).

###### Emended diagnosis.

Males of *P.papilligera* differ from those of other *Paratropis* species by the morphology of the palpal bulb with very thin and long embolus, distally curved (Figure 4F, G), and by the tibia I with a basal retrolateral conic process with spiniform setae (Figure 4D, E).

###### Redescription.

***Male*** (ICN-Ar 2315) (Figure 4): total length 12.3; carapace length 6.1, width 6.8; abdomen length 5.4, width 3.3; chelicerae length 2.7. Color (in alcohol): body with soil particles encrusted; carapace and chelicerae dark reddish brown, legs and abdomen brown (Figure 4A). Carapace slightly setose, striae conspicuous, lateral margins with single line of curved setae mixed with disperse clubbed setae; caput arched, separated from thoracic region by transverse shallow fovea, straight, width 1.0 (Figure 4A). Eyes and ocular tubercle: tubercle length 1.10, width 1.40, very elevated (height 0.9) and forwardly directed, with few stout setae. Clypeus absent. Anterior eye row slightly procurved, posterior recurved. Ocular sizes and interdistances: AME 0.45, ALE 0.35, PME 0.23, PLE 0.33, AME-AME 0.10, AME-ALE 0.08, PME-PME 0.63, PME-PLE 0.05, ALE-PLE 0.08, AME-PME 0.05, ALE-ALE 0.93, PLE-PLE 0.80. Chelicerae: short sparse bristles on dorsal and lateral areas, long fine bristles on ventral and anterior area. Rastellum absent. Cheliceral furrow with two rows of teeth well developed, 10/10 and 9/10 teeth on promargin and retromargin, respectively. Fang long. Labium: length 0.8, width 1.4, with 105 cuspules on anterior edge (Figure 4C). Labio-sternal groove narrower in the middle than laterally. Maxillae longer than wide, with the anterior prolateral lobe very elongated, conical (Figure 4C); with 73/72 rounded cuspules spaced, largely spread over prolatero-ventral border from the inner edge to anterior lobe. Lyra absent. Sternum: length 2.7, width 3.4; three pairs of sigillae, anterior subcircular, median and posterior sigillae oval; anterior and median sigillae marginal, posterior submarginal. Anterior edge of sternum with a semicircular area slightly elevated (joined to labio-sternal groove) (Figure 4C).

Legs: cuticle with soil particles encrusted. Leg and palpal segments measurements provided in Table 4. Leg I clearly thicker than the others. Bristles, plumose and thorn-like setae and spines evident. Tibia I with a basal retrolateral conic process with spiniform setae (Figure 4D, E). Trichobothria: filiform, on central 2/3 of tarsi, palp 6, leg I 9, II 8, III 7, IV 8; on distal 1/4 of metatarsi, leg I 5, II 4, III 4, IV 4; on proximal 1/3 of tibiae, palp two rows of 4 each, leg I two groups of 5 each, II-III two groups of 4 each, IV 2r-5p-1d (proximal/distal). Scopulae absent. Pseudoscopula weak and divided by conical longer setae, only present on distal tarsi I and II; tarsi III and IV with few sparse pseudoscopula setae. Claw tufts absent. Tarsal claws: ITC only present on leg I; STC with one tooth on all legs. Spination: principally thorn-like setae on all segments. Spines: palp 0; leg I 0; leg II 0; leg III, fe 0, pa 0, ti 0, me 1p 1v, ta 1p; leg IV, fe 0, pa 0, ti 0, me 1p, ta 1p.

**Table 4.** Length (in mm) of legs and palpal segments of male *Paratropispapilligera* (ICN-Ar 2315).

	I	II	III	IV	Palp
Femur	7.3	6.2	5.3	7.2	3.0
Patella	3.4	2.7	2.3	2.6	1.8
Tibia	6.5	5.0	4.0	6.1	2.7
Metatarsus	6.2	5.8	5.2	7.2	–
Tarsus	2.2	2.5	2.3	2.9	1.0
Total	25.6	22.2	19.1	26.0	8.5

Palp: cymbium with two unequal lobes separated by a sclerotized groove; tibia with shallow distoventral groove. Palpal bulb elongated; embolus very thin and long (longer than tibia), distally curved, tapering to the apex (Figure 4F, G).

Abdomen: with four longitudinal dorsal rows of seven small tubercles, each emitting from its summit a plumose, bacilliform seta; lateral area finely tuberculate, with smaller plumose setae. Book lung apertures projected, oval, sclerotized (Figure 4B). Spinnerets: PMS length 0.50; PLS length 3.00, apical segment digitiform. Basal segment of PLS divided in two unequal cuticle plates (Figure 4B).

###### Distribution.

Amazonas of Brazil and Colombia. Brazil, Santarem (Pará); Colombia, Amazonas (Leticia) (Figure 10).

##### Stormtropis gen. n.

Taxon classificationAnimaliaAraneaeParatropididae

http://zoobank.org/F0DD1290-6D25-40A3-B9AF-430A4316F24A

**Figure 5.**
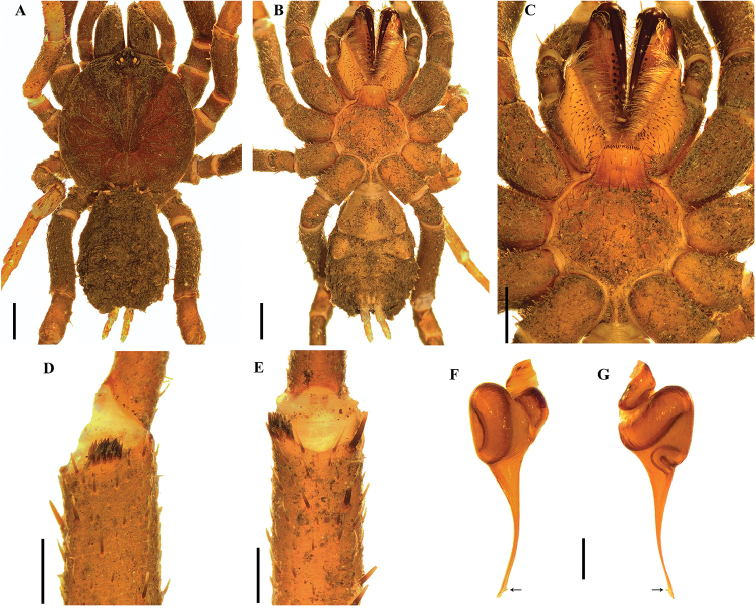
*Stormtropiscolima* gen. n., sp. n., male. **A, B***habitus***A** dorsal view **B** ventral view **C** sternum, labium and maxillae **D, E** tibial apophysis on leg I **D** prolateral view **E** ventral view **F, G** palpal bulb **F** prolateral view **G** retrolateral view. Arrows point to the triangular tooth on the subapical region of the embolus. Scale bars: 1.0 mm (**A–C**); 0.5 mm (**D, E**); 0.25 mm (**F, G**).

**Figure 6.**
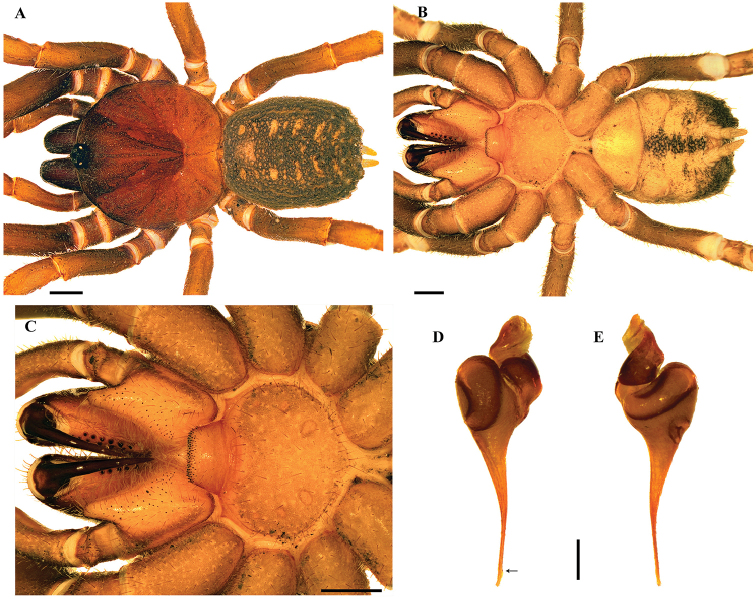
*Stormtropismuisca* gen. n., sp. n., male. **A, B***habitus***A** dorsal view **B** ventral view **C** sternum, labium and maxillae **D, E** palpal bulb **D** prolateral view **E** retrolateral view. Arrows point to the triangular tooth on the subapical region of the embolus. Scale bars: 1.0 mm (**A–C**); 0.25 mm (**D, E**).

**Figure 7.**
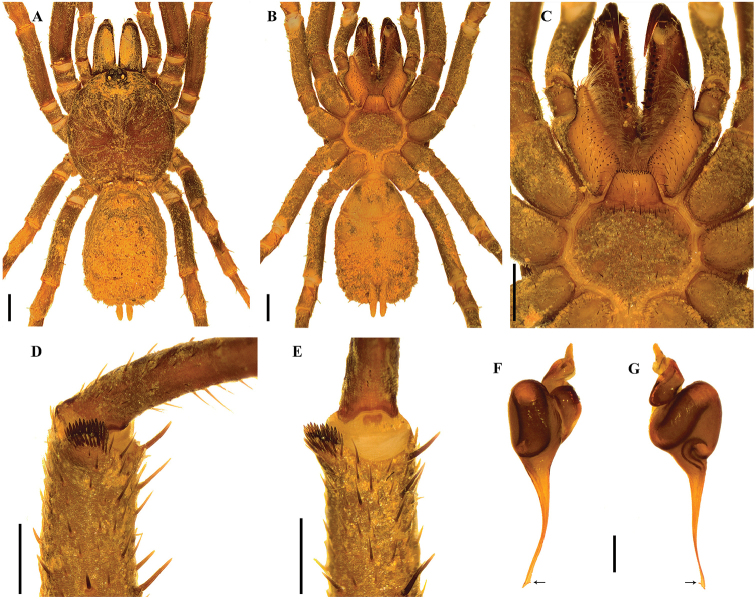
*Stormtropispaisa* gen. n., sp. n., male. **A, B***habitus***A** dorsal view **B** ventral view **C** sternum, labium and maxillae **D, E** tibial apophysis on leg I **D** prolateral view **E** ventral view **F, G** palpal bulb **F** prolateral view **G** retrolateral view. Arrows point to the triangular tooth on the subapical region of the embolus. Scale bars: 1.0 mm (**A–C**); 0.5 mm (**D, E**); 0.25 mm (**F, G**).

**Figure 8.**
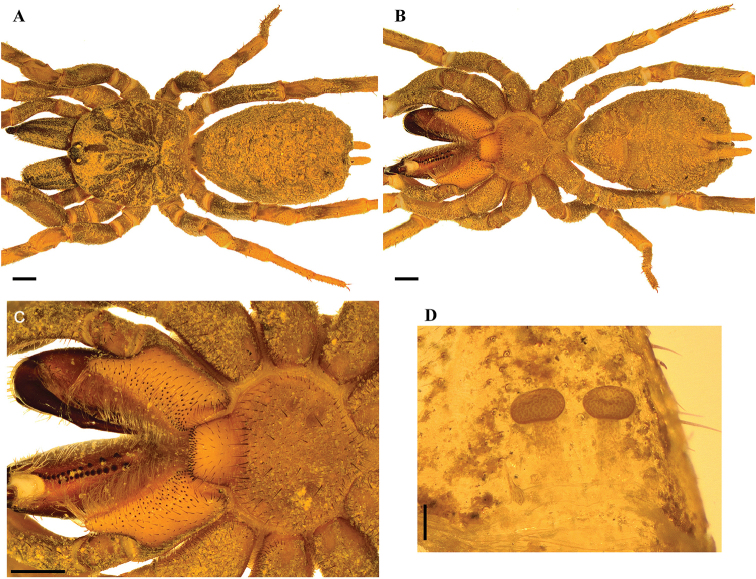
*Stormtropispaisa* gen. n., sp. n., female. **A, B***habitus***A** dorsal view **B** ventral view **C** sternum, labium and maxillae **D** spermathecae. Scale bars: 1.0 mm (**A–C**); 0.2 mm (**D**).

**Figure 9.**
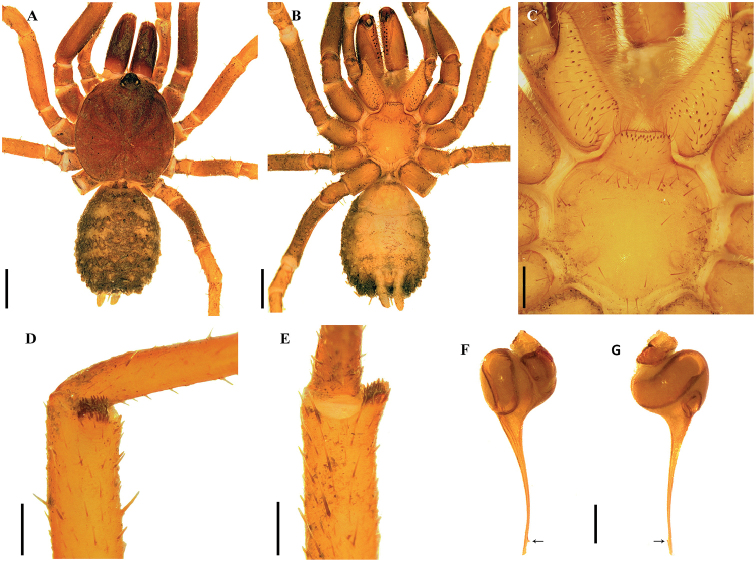
*Stormtropisparvum* gen. n., sp. n., male. **A, B***habitus***A** dorsal view **B** ventral view **C** sternum, labium and maxillae **D, E** tibial apophysis on leg I **D** prolateral view **E** ventral view **F, G** palpal bulb **F** prolateral view **G** retrolateral view. Arrows point to the triangular tooth on the subapical region of the embolus. Scale bars: 1.0 mm (**A, B**); 0.5 mm (**C**); 0.25 mm (**D, E**); 0.25 mm (**F, G**).

Figs 5 , 6 , 7 , 8 , 9

###### Type species.

*Stormtropisparvum* Perafán, Galvis & Pérez-Miles, sp. n.

###### Diagnosis.

*Stormtropis* gen. n. males differ from those of all other paratropidids by the combination of the following characteristics: absence of spines on all segments of legs, lack third claw (ITC) on all tarsi and by the morphology of palpal bulb; pyriform, elongated, with embolus slightly curved tapering to the apex and a subapical triangular tooth (Figs 5F–G, 6D–E, 7F–G, 9F–G). Excepting *S.muisca* sp. n. the other species have a tibial prolateral apophysis, constituted by a single spur and a group of about 15 spines in two parallel rows (Figs 5D–E, 7D–E, 9D–E); it differs from *Melloina* males by the absence of claw tufts and the different morphology of tibial apophysis (two branches and few megaspines in *Melloina*: Schenkel 1953: fig. 4C, D; Raven 1999: fig. 2F; Bertani 2013: figs 12, 13). Females differ from all other paratropidids by the morphology of the spermathecal receptacles with a tubular neck and a wide globose fundus (mushroom shaped) (Figure 8D), and few spines on all legs. Additionally, *Stormtropis* gen. n. has few labial and maxillary elongated cuspules (less than 70), four spinnerets (PMS and PLS), and fewer tricobothriae on each article.

###### Included species.

*Stormtropiscolima* Perafán, Galvis and Pérez-Miles sp. n., *Stormtropismuisca* Perafán, Galvis and Pérez-Miles sp. n., *Stormtropispaisa* Perafán, Galvis and Pérez-Miles sp. n. and *Stormtropisparvum* Perafán, Galvis and Pérez-Miles sp. n.

###### Description.

Carapace round, almost glabrous, light to dark brown. Caput arched. Fovea shallow, transverse, straight to slightly procurved. Eye group subquadrate, wider than long, tubercle well defined, elevated. Clypeus absent. Chelicerae without rastellum, cheliceral furrow narrow with teeth on both margins: promargin 7–13, retromargin 6–13, fangs long. Labium subquadrate with 20–70 cuspules restricted to anterior edge. Maxillae longer than wide with the anterior prolateral lobe very elongated, conical; few cuspules (24–77) throughout the prolateral diagonal half of the maxillae. Labio-sternal groove narrow in the middle and wider laterally. Sternum heart shaped, slightly wider than long, sigillae oval, submarginal. Legs, thin and long, pair I slightly stouter than II-IV; clubbed setae present. Few filiform tricobothria on tarsus, metatarsus, and tibia in males. Long paired claws (STC) with one medial long tooth ventrally; third unpaired claw (ITS) absent on all legs of males; ITS present on leg I of females. Claw tufts absent, tarsal scopula absent, pseudoscopula setae generally present on the distal third of anterior tarsi. Males with spinose apophysis (similar to Aviculariinae) on prolateral distal tibiae I (except *S.muisca*). Abdomen oval, glabrous, with clubbed setae present on dorsum. Four spinnerets; PLS well developed, PMS small (half of the basal segment of PLS). All body encrusted by soil particles. Males without spines, and females with few spines on all legs. Males with cymbium with two unequal lobes separated by a sclerotized groove; palpal tibia with shallow distoventral groove; and palpal bulb pyriform elongated, with embolus slightly curved tapering to the apex, and a subapical triangular tooth. Females with spermathecal receptacles with a tubular neck and globose fundus.

###### Distribution.

**Figure 10.**
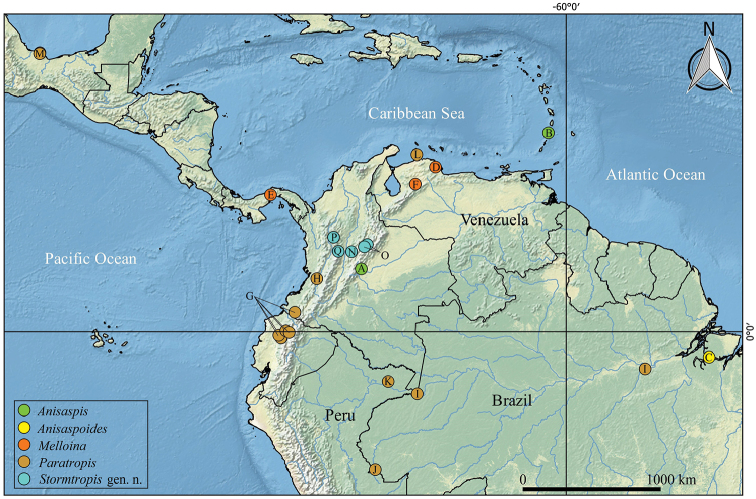
Distribution records of the entire family Paratropididae Simon, 1889; genera differentiated by color. **A***Anisaspiscamarita* sp. n. **B***Anisaspistuberculata* Simon, 1892 **C***Anisaspoidesgigantea* F.O. Pickard-Cambridge, 1896 **D***Melloinagracilis* (Schenkel, 1953) **E***Melloinarickwesti* Raven, 1999 **F***Melloinasantuario* Bertani, 2013 **G***Paratropiselicioi* Dupérré, 2015 **H***Paratropisflorezi* sp. n. **I***Paratropispapilligera* F.O. Pickard-Cambridge, 1896 **J***Paratropissanguinea* Mello-Leitão, 1923 **K***Paratropisscruposa* Simon, 1889 **L***Paratropisseminermis* Caporiacco, 1955 **M***Paratropistuxtlensis* Valdez-Mondragón, Mendoza & Francke, 2014 **N***Stormtropiscolima* gen. n., sp. n. **O***Stormtropismuisca* gen. n., sp. n. **P***Stormtropispaisa* gen. n., sp. n. **Q***Stormtropisparvum* gen. n., sp. n.

*Stormtropis* gen. n. is distributed in the central and eastern Cordilleras of Colombia, on the montane forests of the Magdalena Valley and Cauca Valley, between 1400–3400 m altitudes, in the Departments of Antioquia (Santa Elena), Boyacá (Sotaquirá and Santuario de Fauna y Flora Iguaque), Caldas (Pensilvania) and Cundinamarca (Topaipí) (Figure 10).

###### Etymology.

The name *Stormtropis* is a Latin declension (neuter) of the noun Stormtrooper from the fictional universe of the Star Wars films. The stormtroopers are the soldiers of the main ground force of the Galactic Empire. These soldiers are very similar to each other, with some capacity for camouflage but with unskillful movements, like this group of spiders.

##### Stormtropis colima sp. n.

Taxon classificationAnimaliaAraneaeParatropididae

http://zoobank.org/B0A52980-1C2D-4CA5-A813-B0BCC6806314

Figure 5

###### Type material.

***Holotype*** male from Colombia, Cundinamarca, Río Negro Province, Topaipí, 1377 m, 18-23-x-2012, col. M Medrano, A García, E Martínez (ICN-Ar 11439).

###### Diagnosis.

*Stormtropiscolima* sp. n. differs from the other species of the genus by the presence of a tibial apophysis with shorter base and not so much separated from the tibia as in the other species (Figure 5D, E). Additionally, *S.colima* sp. n. differs from *S.parvum* sp. n. by the presence of a continuous row and more numerous cheliceral teeth on promargin (13–11) (7 in *S.parvum* sp. n.) and from *S.paisa* sp. n. by the absence of a sclerotized dark mark on proximal dorsal tibia, without a slight excavation.

In addition, *S.colima* sp. n. is larger (8.4 mm) and lives at lower elevation (1377m) than *S.parvum* sp. n. (6 mm; 2750 m) and *S.paisa* sp. n. (8.5 mm; 2400 m).

###### Description.

***Holotype male*** (ICN-Ar 11439) (Figure 5): total length 8.4, carapace length 4.2, width 4.2; abdomen length 3.3, width 2.7; chelicerae length 1.7. Color (in alcohol): body with soil particles encrusted; carapace, chelicerae, coxa, trochanter and femur dark brown; abdomen dorsally and patella-tarsus brown. Carapace: glabrous, striae conspicuous, lateral margins with single line of curved setae mixed with disperse clubbed setae; caput arched, separated from thoracic region by transverse shallow fovea, straight, width 0.4 (Figure 5A). Eyes and ocular tubercle: tubercle length 0.6, width 0.7, very elevated (height 0.5) and forwardly directed, with few stout setae. Clypeus absent. Anterior eye row slightly procurved, posterior recurved. Ocular sizes and interdistances: AME 0.23, ALE 0.25, PME 0.15, PLE 0.18, AME-AME 0.08, AME-ALE 0.03, PME-PME 0.28, PME-PLE 0.03, ALE-PLE 0.05, AME-PME 0.03, ALE-ALE 0.38, PLE-PLE 0.45. Chelicerae: dorsal few plumose setae, short sparse bristles on dorsal and lateral areas, long fine bristles on ventral and anterior areas. Rastellum absent. Cheliceral furrow narrow with two rows of teeth, 13/11 teeth on promargin, and 8 teeth on retromargin. Fang long. Labium subquadrate, length 0.5, width 1.00, with 39 elongated cuspules on anterior edge (Figure 5C). Labio-sternal groove narrower in the middle than laterally. Maxillae longer than wide, with the anterior prolateral lobe very elongated, conical; with 42/44 elongated cuspules widely distributed throughout the prolateral half of the maxillae; field of cuspules wider proximally (Figure 5C). Sternum heart shaped, length 1.73, width 2.15; three pairs of sigillae, anterior and median subcircular, posterior sigillae oval, all marginal (Figure 5C).

Legs: cuticle with soil particles encrusted. Leg and palpal segments measurements provided in Table 5. Legs I slightly stouter than II–IV. Femur IV widened. Clubbed plumose and thorn-like setae. Trichobothria: filiform, on central 2/3 of tarsi, palp, and all legs 5; on distal 1/4 of metatarsi, all legs 3; and on proximal 1/3 of tibiae, palp and legs I–III two rows of 2 each, IV 4. Scopula absent, few and sparse pseudoscopula setae on tarsi I and II. Claw tufts absent. Tarsal claws: ITC absent from all legs; STC long, with one tooth on all legs. Tibial apophysis on leg I present: a short flattened branch on distal prolateral side, with spines on two parallel rows, 10 distal and 5 proximal (Figure 5D, E). Thorn-like setae mainly present on legs III and IV. Spines absent.

**Table 5.** Length (in mm) of legs and palpal segments of the holotype male *Stormtropiscolima* sp. n.

	I	II	III	IV	Palp
Femur	3.9	3.2	2.7	3.6	1.7
Patella	1.9	1.5	1.3	1.5	1.2
Tibia	3.1	2.3	1.7	3.0	1.3
Metatarsus	3.0	2.5	2.4	3.5	–
Tarsus	1.5	1.4	1.4	1.7	0.6
Total	13.4	10.9	9.5	13.3	4.8

Palp: cymbium with two unequal lobes separated by a sclerotized groove; tibia with shallow distoventral groove. Palpal bulb pyriform elongated; embolus curved, very long, tapering to the apex, apex wide; a triangular translucent tooth on subapical region, close to apex (Figure 5F, G).

Abdomen: oval, with small clubbed setae; lateral and dorsal areas finely tuberculate, with small plumose clubbed setae, principally on posterior area (Figure 5A). Book lung apertures projected, oval, sclerotized (Figure 5B). Spinnerets: PMS length 0.3; PLS length 1.6, apical segment digitiform (Figure 5B).

***Female***: unknown.

###### Distribution.

Only known from its type locality, Topaipí in the Río Negro Province (Cundimarca), in the Eastern Cordillera of Colombian Andes, at 1300 m altitude (Figure 10).

###### Etymology.

The species epithet *colima* is a noun in apposition which means warrior in the extinct Muisca language. The Colimas were an indigenous tribe that inhabited in the central highlands of Colombia, where the species occurs.

##### Stormtropis muisca sp. n.

Taxon classificationAnimaliaAraneaeParatropididae

http://zoobank.org/BDD00B70-6EDF-480B-9CAA-87FE0C174A85

Figure 6

###### Type material.

***Holotype*** male from Colombia, Boyacá, Sotaquirá, Vereda Guaguaní, 5.80886N, 73.25063W, 3415 m, 8-10-vi-2015, col. Y Cifuentes, J Moreno (ICN-Ar 11440). ***Paratype***: 1 male from Colombia, Boyacá, Villa de Leyva, Santuario de Flora y Fauna Iguaque, 11-iv-2000, pitfall, col. C Fagua (ICN-Ar 880).

###### Diagnosis.

Males of *Stormtropismuisca* sp. n. differ from those of all other species of *Stormtropis* gen. n. by the absence of tibial apophysis, and by the pattern of abdominal color, grayish brown with a pattern of seven pairs of lighter dots, and sub-circular carapace (Figure 6A). Also, by the palpal bulb elongated, with the embolus slightly curved tapering to the apex, which is stout but flattened, and a very small subapical triangular tooth (Figure 6D, E).

###### Description.

***Male*** (ICN-Ar 11440) (Figure 6): total length 9.5; carapace length 4.6, width 4.8; abdomen length 4.6, width 3.5; chelicerae length 1.3. Color (in alcohol): body with few soil particles encrusted; carapace, chelicerae and legs dark reddish brown; abdomen grayish brown with a pattern of seven pairs of lighter dots (Figure 6A). Carapace slightly setose, striae conspicuous, lateral margins with single line of spiniform setae; caput very arched, separated from thoracic region by transverse shallow fovea, straight, width 0.9 (Figure 6A). Eyes and ocular tubercle: tubercle length 0.73, width 0.8, very elevated (height 0.5) and forwardly directed, with few stout setae. Clypeus absent. Anterior eye row slightly procurved, posterior recurved. Ocular sizes and interdistances: AME 0.25, ALE 0.25, PME 0.10, PLE 0.20, AME-AME 0.15, AME-ALE 0.25, PME-PME 0.40, PME-PLE 0.03, ALE-PLE 0.08, AME-PME 0.05, ALE-ALE 0.50, PLE-PLE 0.50. Chelicerae: short sparse bristles on dorsal and lateral areas, long fine bristles on ventral and anterior areas. Rastellum absent. Cheliceral furrow with two rows of well-developed teeth, 8/7 and 6/6 teeth on promargin and retromargin, respectively. Labium: length 0.5, width 1.2, with 69 cuspules on anterior edge (Figure 6C). Labio-sternal groove of uniform width. Maxillae longer than wide, with the anterior prolateral lobe very elongated, conical (Figure 6C); with 37/36 conical cuspules spaced, largely spread over prolatero-ventral border from the inner edge to anterior lobe. Lyra absent. Sternum: length 1.8, width 2.5; three pairs of sigillae, anterior subcircular, median and posterior sigillae oval; anterior and median sigillae marginal, posterior submarginal. Anterior edge of sternum with a semicircular area slightly elevated (joined to labio-sternal groove) (Figure 6C).

Legs: cuticle normal. Leg and palpal segments measurements provided in Table 6. Leg I clearly thicker than the others. Bristles and thorn-like setae present. Trichobothria: filiform, on central 2/3 of tarsi, palp 4, leg I 7, II 5, III 4, IV 4; on distal 1/4 of metatarsi, leg I 4, II 3, III 3, IV 3; on proximal 1/3 of tibiae, palp and legs I-III two rows of 2 each, IV a row of 3p and 1d. Scopulae absent. Pseudoscopula weak and divided by conical longer setae, only present on distal tarsi I, II and III; tarsi IV with few sparse pseudoscopula setae. Claw tufts absent. Tarsi distally incrassate, mainly on anterior legs. Tarsal claws: ITC absent on all legs, but a very small tooth present on legs I; STC with one curved tooth on all legs. Spination: principally thorn-like setae on all segments. Spines absent.

**Table 6.** Length (in mm) of legs and palpal segments of the holotype male *Stormtropismuisca* sp. n.

	I	II	III	IV	Palp
Femur	4.0	3.5	3.0	3.8	1.9
Patella	2.3	1.8	1.4	1.8	1.4
Tibia	3.5	2.6	2.0	3.1	1.4
Metatarsus	3.0	2.7	2.2	3.0	–
Tarsus	1.6	1.5	1.5	1.5	0.9
Total	14.4	12.1	10.1	13.2	5.6

Palp: cymbium with two unequal lobes separated by a sclerotized groove; tibia with shallow distoventral groove. Palpal bulb pyriform; embolus slightly curved tapering to the apex, apex stout but flattened, a subapical triangular tooth on embolus (Figure 6D, E).

Abdomen: with four longitudinal dorsal rows of nine small tubercles, each emitting from its summit a spiniform seta. Book lung apertures projected, oval, sclerotized (Figure 6B). Spinnerets: PMS length 0.10; PLS length 1.40, apical segment digitiform (Figure 6B).

***Female***: Unknown.

###### Distribution.

Eastern Cordillera of Colombian Andes, at a height above 3000 m, Páramo biogeographic province. Boyacá Department, municipality of Sotaquirá (Guaguaní) and Iguaque Fauna and Flora Sanctuary (Figure 10).

###### Etymology.

The species epithet *muisca* is a noun in apposition which refers to the indigenous tribe who inhabit in the same region where this species occur.

##### Stormtropis paisa sp. n.

Taxon classificationAnimaliaAraneaeParatropididae

http://zoobank.org/BDCF8B14-4E67-436B-9BE8-7625D492002A

Figures 7 , 8

###### Type material.

***Holotype*** male from Colombia, Antioquia, Santa Elena, Parque Ecoturístico Arví, Piedras Blancas, 12-iv-2017, 2400 m, col. C. Perafán, L. Montes de Oca, F. Pérez-Miles, J. Salazar, (ICN-Ar 11441). ***Paratypes***: 3 females with the same data (ICN-Ar 11442-11443) (FC-My 1409).

###### Diagnosis.

*Stormtropispaisa* sp. n. differs from the other species of the genus by the presence of a sclerotized dark mark on proximal dorsal tibia, with a slight excavation (Figure 7A). Males additionally differ from the other species by the presence of a tibial apophysis bearing more numerous spines on the proximal row (12) (Figure 7D, E); in the other species with tibial apophysis the number of such spines is 5–6; and by the palpal bulb with the embolus sinuous, distally twisted and more widened on the apex (Figure 7F, G). Females can be distinguished by the morphology of the spermathecal receptacles with a tubular neck and a wide globose fundus (mushroom shaped) (Figure 8D).

###### Description.

***Holotype male*** (ICN-Ar 11441) (Figure 7): total length 8.5; carapace length 3.9, width 4.0; abdomen length 4.7, width 2.7; chelicerae length 1.6. Color (in alcohol): body with soil particles encrusted; carapace, chelicerae, coxa, trochanter and femur reddish brown; abdomen dorsally and patella-tarsus brown, tibiae dorsally with a proximal dark brown mark. Carapace: glabrous, striae conspicuous, lateral margins with single line of curved setae mixed with dispersed clubbed setae; caput arched, with three longitudinal lines of plumose setae; caput slightly arched, separated from thoracic region by transverse shallow fovea, straight, width 0.7 (Figure 7A). Eyes and ocular tubercle: tubercle length 0.6, width 0.8, very elevated (height 0.6) and forwardly directed, with few stout setae. Clypeus absent. Anterior eye row slightly procurved, posterior slightly recurved. Ocular sizes and interdistances: AME 0.25, ALE 0.30, PME 0.20, PLE 0.25, AME-AME 0.10, AME-ALE 0.03, PME-PME 0.35, PME-PLE 0.03, ALE-PLE 0.05, AME-PME 0.03, ALE-ALE 0.50, PLE-PLE 0.48. Chelicerae: short sparse bristles on dorsal and lateral areas, long bristles on ventral and anterior area. Rastellum absent. Cheliceral furrow narrow, with two rows of teeth, 10 teeth on promargin, 10/9 teeth on retromargin. Fang long. Labium sub-trapezoid, length 0.90, width 0.50, with 33 elongated cuspules on anterior edge (Figure 7C). Labio-sternal groove wide. Maxillae longer than wide, with the anterior prolateral lobe very elongated, conical; with 35 elongated cuspules widely distributed throughout the prolateral half of the maxillae; field of cuspules wider proximally (Figure 7C). Sternum heart shaped with an anterior nodule, length 1.60, width 2.00; three pairs of sigillae, anterior and median subcircular, submarginal; posterior sigillae oval, marginal (Figure 7C). Anterior edge of sternum with a semicircular area slightly elevated (joined to labio-sternal groove) (Figure 7C).

Legs: cuticle with soil particles encrusted. Leg and palpal segments measurements provided in Table 7. Legs I slightly stouter than II-IV. Clubbed plumose and thorn-like setae. Trichobothria: filiform, on central 2/3 of tarsi, palp and all legs 5; on distal 1/4 of metatarsi, leg I 4, II–IV 3; on proximal 1/3 of tibiae, palp and legs I-III 5 in two rows (2/3 each), IV 4. Scopula absent, pseudoscopula slightly denser on tarsi I and II, sparse pseudoscopula setae on tarsi III and IV. Claw tufts absent. Tarsal claws: ITC absent on all legs; STC long, with one tooth on all legs. Tibial apophysis on leg I present: only one flattened branch on distal prolateral side, with spines on two parallel rows, 15 distal and 12 proximal (Figure 7D, E). Thorn-like setae on tibiae, metatarsi, and tarsi of all legs, more dense on legs III and IV. Spines absent.

**Table 7.** Length (in mm) of legs and palpal segments of the holotype male / paratype female *Stormtropispaisa* sp. n.

	I	II	III	IV	Palp
Femur	3.8/3.8	3.1/3.0	2.6/2.7	3.5/3.7	1.8/2.2
Patella	1.8/2.2	1.5/1.6	1.3/1.5	1.4/1.6	1.1/1.3
Tibia	3.0/2.8	2.2/1.9	1.7/1.6	2.8/2.7	1.3/1.2
Metatarsus	2.8/2.3	2.4/2.0	2.1/2.0	3.1/3.1	–
Tarsus	1.6/1.3	1.5/1.3	1.3/1.3	1.7/1.7	0.8/1.9
Total	13.0/12.4	10.7/9.8	9.0/9.1	12.5/12.8	5.0/6.6

Palp: cymbium with two unequal lobes separated by a sclerotized groove; tibia with a distoventral groove. Palpal bulb pyriform elongated; embolus sinuous and distally twisted, long, tapering to the apex; a triangular translucent tooth on subapical region (Figure 7F, G).

Abdomen: oval, with seven transverse dorsal rows of 4–6 small clubbed setae; with smaller plumose clubbed setae, principally on posterior area (Figure 7A). Book lung apertures projected, oval, and sclerotized (Figure 7B). Spinnerets: PMS length 0.30; PLS length 1.4, apical segment digitiform (Figure 7B).

***Female*** (ICN-Ar 11442) (Figure 8): total length 11.7; carapace length 4.8, width 4.6; abdomen length 6.4, width 4.0; chelicerae length 2.4. Color, coverage, and habitus as in male (Figure 8A, B). Eyes and ocular tubercle: tubercle length 0.7, width 1.0, very elevated (height 0.7) and forwardly directed, with setae. Clypeus absent. Anterior eye row slightly procurved, posterior recurved. Ocular sizes and interdistances: AME 0.25, ALE 0.33, PME 0.23, PLE 0.25, AME-AME 0.05, AME-ALE 0.05, PME-PME 0.43, PME-PLE 0.03, ALE-PLE 0.05, AME-PME 0.05, ALE-ALE 0.55, PLE-PLE 0.60. Chelicerae as in male. Rastellum absent. Cheliceral furrow with two rows of well-developed teeth, 12/7 teeth on promargin, 11/13 teeth on retromargin. Labium sub-trapezoid, length 0.6, width 1.2, with 53 cuspules on anterior edge (Figure 8A). Labio-sternal groove wide. Maxillae longer than wide, with the anterior prolateral lobe very elongated, conical; with 75/77 cuspules spaced, largely spread over prolatero-ventral border from the inner edge to anterior lobe. Sternum and sigillae as in male; sternum length 2.0, width 2.7 (Figure 8A).

Legs: cuticle as in male. Leg and palpal segments measurements provided in Table 7. Legs I clearly thicker than the others. Bristles, clubbed, thorn-like setae, and spines present. Trichobothria: filiform, on central 2/3 of tarsi, palp 5, legs I–II 5, III 4, IV 6; on distal 1/4 of metatarsi, leg I 4, II 3, III 4, IV 2; on proximal 1/3 of tibiae, palp 4, legs I-II two rows of 2 each, III two rows of 3 and 2 each, IV 4. Scopula and pseudoscopula absent. Claw tufts absent. Tarsal claws: ITC present on leg I; STC present on all legs with one tooth. Spination: principally thorn-like setae on all segments. Spines: palp 0; leg I fe 0, pa 0, ti 0, me 2v, 2pv, 4rv,1r, ta 4pv, 5rv; leg II fe 0, pa 0, ti 0, me 1pd, 2v, 1pv, ta 0; leg III fe 0, pa 0, ti 0, me 1pv, 1pd, ta 0; leg IV fe 0, pa 0, ti 0, me 1pv, ta 0.

Abdomen: book lung apertures and spinnerets as in male (Figure 8B). Two spermathecal receptacles with a tubular neck, ended in a globose flattened fundus; spermathecal fundus with higher density of glands than the neck (mushroom shaped, Figure 8D). Spinnerets: PMS length 0.40; PLS length 2.20, apical segment digitiform (Figure 8B).

###### Distribution.

Only known from its type locality, Central Cordillera of the Colombian Andes, Antioquia Department, Medellin (Santa Elena), at 2400 m altitude (Figure 10).

###### Etymology.

The species epithet *paisa* is a noun in apposition which means the vernacular name given to the people of Medellín, where the species occurs.

##### Stormtropis parvum sp. n.

Taxon classificationAnimaliaAraneaeParatropididae

http://zoobank.org/0D2EEF63-4E0D-4D0E-A067-F179CC4D45D5

Figure 9

###### Type material.

***Holotype*** male from Colombia, Caldas, Pensilvania, Berlín, 5.35222N, 75.18611W, 2750 m, 24-28-vii-2004, col. E Gonzáles, L Arango, JM Molina (ICN-Ar 11444).

###### Diagnosis.

*Stormtropisparvum* sp. n. differs from the other species of the genus by the less numerous and discontinuous row of cheliceral teeth on promargin (2-2-3) (Figure 9B), and by the presence of a tibial apophysis, with a larger base, more separated from the tibia than on the other species (Figure 9D, E). Additionally, differs from *S.paisa* by the absence of a sclerotized dark mark on proximal dorsal tibia, without excavation, and lower number of spines in the spur (eleven on the distal row and six on proximal).

In addition, *S.parvum* is smaller (6mm), with lighter red and brown tones, and lives at higher elevation (2750 m) than *S.colima* (8.4 mm; 1377 m) and *S.paisa* (8.5 mm; 2400 m).

###### Description.

***Holotype male*** (ICN-Ar 11444) (Figure 9): total length 6.0; carapace length 2.9, width 2.8; abdomen length 2.8, width 2.3; chelicerae length 1.5. Color (in alcohol): body with soil particles encrusted; carapace, chelicerae, coxa, trochanter and femur red brown; abdomen dorsally and patella-tarsus brown. Carapace: glabrous, striae conspicuous, lateral margins with single line of curved setae mixed with disperse clubbed setae; caput arched, separated from thoracic region by transverse shallow fovea, straight, width 0.3 (Figure 9A). Eyes and ocular tubercle: tubercle length 0.5, width 0.6, very elevated (height 0.4) and forwardly directed, with few stout setae. Clypeus absent. Anterior eye row slightly procurved, posterior slightly recurved. Ocular sizes and interdistances: AME 0.15, ALE 0.18, PME 0.13, PLE 0.14, AME-AME 0.10, AME-ALE 0.03, PME-PME 0.25, PME-PLE 0.03, ALE-PLE 0.03, AME-PME 0.03, ALE-ALE 0.35, PLE-PLE 0.38. Chelicerae: dorsally with few plumose setae, short sparse bristles on dorsal and lateral areas, long fine bristles on ventral and anterior area. Rastellum absent. Cheliceral furrow narrow with two rows of teeth, 7 teeth on promargin, on a discontinuous row (proximal-distal: 2-2-3); and 8/9 teeth on retromargin. Fang long. Labium subquadrate, length 0.25, width 0.65, with 20 elongated cuspules on anterior edge (Figure 9C). Labio-sternal groove narrower in the middle than laterally. Maxillae longer than wide, with the anterior prolateral lobe very elongated, conical; with 24/29 elongated cuspules widely distributed throughout the prolateral half of the maxillae; field of cuspules wider proximally (Figure 9C). Sternum heart shaped, length 1.15, width 1.53; three pairs of sigillae, anterior and median subcircular, submarginal; posterior sigillae oval, marginal (Figure 9C).

Legs: cuticle with soil particles encrusted. Leg and palpal segments measurements provided in Table 8. Leg I slightly stouter than II–IV. Clubbed plumose and thorn-like setae. Trichobothria: filiform, on central 2/3 of tarsi, palp and all legs 3; on distal 1/4 of metatarsi, all legs 2; and on proximal 1/3 of tibiae, palp and legs I–III two rows of 2 each, IV 3. Scopula absent, few and sparse pseudocopula setae on tarsi I and II. Claw tufts absent. Tarsal claws: ITC absent on all legs; STC long, with one tooth on all legs. Tibial apophysis on leg I present: only one flattened branch on distal prolateral side, with spines on two parallel rows: 11 distal and 6 proximal (Figure 9D, E). Thorn-like setae mainly present on legs III and IV. Spines absent.

**Table 8.** Length (in mm) of legs and palpal segments of the holotype male *Stormtropisparvum* sp. n.

	I	II	III	IV	Palp
Femur	2.6	2.1	1.8	2.5	1.4
Patella	1.3	1.1	0.9	1.0	0.8
Tibia	2.1	1.5	1.3	2.0	1.0
Metatarsus	1.9	1.7	1.5	2.1	–
Tarsus	1.1	1.1	1.1	1.2	0.5
Total	9.0	7.5	6.6	8.8	3.7

Palp: cymbium with two unequal lobes separated by a sclerotized groove; tibia with shallow distoventral groove. Palpal bulb pyriform elongated; embolus curved very long, tapering to the apex; a triangular translucent tooth on the subapical region (Figure 9F, G).

Abdomen: oval, with seven transverse dorsal rows of 4–6 small clubbed setae; lateral and dorsal area finely tuberculate, with smaller plumose clubbed setae, principally on posterior area (Figure 9A). Book lung apertures projected, oval, sclerotized (Figure 9B). Spinnerets: PMS length 0.20; PLS length 1.10, apical segment digitiform (Figure 9B).

***Female***: unknown.

###### Distribution.

Only known from its type locality, in the Central Cordillera of Colombian Andes, eastern flank, at 2750 m altitude, Caldas Department, Pensilvania, Berlín (Figure 10).

###### Etymology.

The species epithet *parvum* is a Latin adjective (neuter) which means little; *S.parvum* sp. n. is the smallest species of the genus known to date.

## Discussion

Our examination of collections as well as field work revealed that paratropidids are very well represented among the aracnofauna of Colombia, currently represented by three genera and eight species. They are commonly present in diverse habitats, mainly in the soil, under stones, rotten logs and in burrows or crevices in ravines. Unexpectedly, and in spite of few unpublished data, the family was not formally reported for Colombia until now. However, de Mello-Leitão (1941) recorded *Paratropisscruposa* Simon, 1889 for Cúcuta (Colombia): this determination has not been included in the World Spider Catalog (2018); possibly this is a wrong identification, since the type locality of this species is in Peru. In this way, Paratropididae is here officially reported for Colombia as well as *Anisaspis*, *Paratropis*, and a new genus, *Stormtropis*.

*Stormtropis* gen. n. is described and represented only in Colombia, by *S.colima* sp. n., *S.muisca* sp. n., *S.paisa* sp. n., and *S.parvum* sp. n. The genus is distributed in the central Andean region of the country, between altitudes of 1377 and 3415 m. The altitudinal record of *S.muisca* sp. n. (above 3400 m) represents the highest of the family Paratropididae. *Anisaspis* is represented by *A.tuberculata* (St. Vincent) and *A.camarita* sp. n. (Colombia); the latter is distributed in the foothills of the eastern Colombian Andes, ca. 570–600 m altitude. *Paratropis* is distributed in Mexico, Antilles, and northern South America. It is represented by seven species, one of them described here as *P.florezi* sp. n., that inhabits in the south of western Cordillera of the Colombian Andes, at ca. 1800 m altitude. The geographic distributions of *P.elicioi* and *P.papilligera* are extended; the first to the south of the Colombian Andes and the second in the Colombian Amazon. *Paratropis* is widely distributed throughout Colombia and Ecuador and is present in almost all regions, from coasts and Amazonian lowlands reaching heights of ca. 2000 m in the Andean region. We have also records of the presence of the genus *Melloina* in Colombia, but we did not include it here because we have molecular evidence that places it out of Paratropididae (Perafán et al. in preparation).

Although Colombian paratropidid species richness is the highest found in a country (WSC 2018), several undescribed species are expected to be found in the future, considering the enormous and unexplored ecological diversity of mygalomorph spiders in this region (Perafán 2017, Perafán and Valencia-Cuéllar 2018), coupled with the evidence of numerous specimens not yet studied.

*Stormtropis* gen. n. includes some species in which males have a tibial apophysis, a feature not found among paratropidids (except *Melloina*) until now. It is probably a recent acquisition of this group of species, which should be more closely related with *Paratropis*, but this hypothesis must be tested with a rigorous phylogenetic analysis. Another remarkable morphological characteristic of this genus is the sexual dimorphism in tarsal claws: ITC on leg I is only present in females. The presence of ITC on leg I was originally used to diagnose *Paratropis* from other paratropidids, but Valdéz-Mondragón et al. (2014) found that females of *Paratropistuxtlensis* have ITC on legs I and II but males lack them. Likewise, Dupérré (2015) described *Paratropiselicioi*, lacking ITC in both sexes, and considered this character as ambiguous. We consider that there is a great inter- and intraspecific variation in this character, so this independent character should be carefully taken into account in the taxonomy of the family. Another sexual difference found in the Paratropidinae is related to the pseudoscopula, present in males and absent in females, as was reported for several Mygalomorphae (Pérez-Miles et al. 2017).

An important characteristic of Paratropididae, unique among Mygalomorphae, is the ability to adhere soil particles to their scaly cuticle. Although the natural history of the group is poorly known, paratropidids were considered cursorial spiders which hide themselves in the surface layers of the soil (West in Raven 1999). This condition should be compatible with the presence of soil encrusted on the cuticle, facilitating the camouflage of individuals on the ground. However, and unexpectedly in our field study, we found several females of *P.florezi* sp. n. and other undetermined species inhabiting tubular burrows on ravine walls or soil. Considering that burrowing paratropidids maintain the encrusted cuticle, burrowing habits could be a secondary adaptation in order to exploit different habitats.

## Supplementary Material

XML Treatment for Anisaspis

XML Treatment for Anisaspis camarita

XML Treatment for Paratropis

XML Treatment for Paratropis elicioi

XML Treatment for Paratropis florezi

XML Treatment for Paratropis papilligera

XML Treatment for Stormtropis

XML Treatment for Stormtropis colima

XML Treatment for Stormtropis muisca

XML Treatment for Stormtropis paisa

XML Treatment for Stormtropis parvum
